# Emerging industrial clusters of disaster safety industry in Korea

**DOI:** 10.1016/j.heliyon.2023.e17939

**Published:** 2023-07-05

**Authors:** Min-Kyu Kim, Jungseok Seo, NaYoon Kim, Ji-Bum Chung

**Affiliations:** Ulsan National Institute of Science and Technology, 50, UNIST-gil, Ulsan 44919, Republic of Korea

**Keywords:** Disaster safety industry, Business network analysis, Emerging industry, Industrial cluster, Korea

## Abstract

Disaster-related industries have become essential in strengthening both disaster resilience and national competitiveness. For more efficient disaster management, the Korean government widely integrated the disaster industry with the safety industry in 2013, calling it the “disaster safety industry.” This study examines the spatial characteristics of the disaster safety industry and its association with regional industries. In emerging industries such as disaster safety industry, there is a scarcity of information regarding intra-industry transactions, and the industry's scope is often vague, thereby restricting comprehensive analysis. To address this issue, we constructed a quasi-business transaction network that aggregates firm level data to regional level. A correlation analysis using location quotients (LQ) was conducted to determine the relationship with the existing industry. The disaster safety industry network was highly correlated with regional demand. The cluster analysis results showed that four clusters were derived around large cities in the region, which was statistically significant. As a result, these cluster formations were statistically significantly correlated with science- and technology-related industries. Although the disaster safety industry was fostered by the government, we confirmed that technological innovation based on existing industries related to science-based technology can also promote the development of the disaster safety industry.

## 1 Introduction

Internationally, discussions on fostering disaster-related industries as emerging industries are underway. As large-scale disasters frequently occur worldwide and uncertainties have increased, governments can hardly deal with such large disasters alone. With the emergence of the concept of resilience in disaster management, this field has gradually expanded from government-led to being led by various stakeholders such as the private companies [1]. Stronger public and private partnerships (PPPs) have been highly emphasized in the international organizations related with disaster risk reduction [2]. The United Nations Office for Disaster Risk Reduction (UNDRR) has stressed that current disaster management needs whole-society engagement and emphasizes the role of the private sector [2]. Indeed, private companies can play crucial roles in enhancing disaster resilience at the local level by providing disaster prevention and safety-related products and services through cooperation with governments [[2], [3], [4]].

An important role of the government is to strengthen national competitiveness by accurately identifying and fostering industries. Government support and management are essential for emerging industries that are socially important but in the early stages of development. Recognition and a better understanding of emerging industries for future growth are vital for effective policy development [5,6]. The emerging industry can result from existing technologies and solutions entering new application fields or a combination of new and previous technologies [7]. Porter [8] argued that emerging industries are newly formed or reformed industries created by technological innovation, the needs of new consumers (including public sectors), and economic and sociological changes. From the viewpoint of “technology push,” innovative technology development has the potential to increase the performance and productivity of existing industrial sectors by enabling the production of new products and services, as well as the reconstruction of existing industrial processes [6,9]. Meanwhile, from the viewpoint of “market pull,” some emerging industries can evolve due to market demand, including the government and public sectors. The unique Korean “disaster safety industry” can be characterized as an emerging industry that has been developed according to the government's needs.

Emerging industries tend to be spatially integrated for innovation and creative opportunities [10], which comes with cluster growth [[11], [12], [13]]. The emergence of new industries is influenced by clusters that provide an advantageous business environment from the early stages of formation. These clusters are formed at the beginning of the lifecycle stage and create competition and cooperation between companies from different industrial backgrounds. Emerging industries exhibit uncertainty and small-scale characteristics during the early stages of their life cycle [7], which makes detecting technological innovation and identifying emerging clusters a challenging task [14]. Nevertheless, recognizing cluster formation within emerging industries at the onset of their life cycle is a crucial initial step in understanding and promoting the development of such industries [5].

The Korean government is actively promoting the advancement of the disaster safety industry through a multitude of initiatives. It has set up a robust legal framework with measures such as the Disaster and Safety Management Technology Development Comprehensive Plan and the Disaster and Safety Industry Promotion Act. Furthermore, regional clusters are considered a feasible strategy to spur the growth of the disaster safety industry, and corresponding national and regional policies are being devised [15]. Given these circumstances, the research problem addressed in this study aims to investigate whether the disaster safety industry exhibits a regional cluster pattern as an emerging industry and to identify its spatial characteristics. Through this analysis, we will explore the potential of regional clusters for advancing the disaster safety industry. Specifically, our research addresses following research questions: 1) what are the spatial characteristics of the disaster safety industry as clusters? and 2) how do the clusters relate to existing industry characteristics?

To answer these research questions, we constructed an ‘input-output’ (I–O) relationship based quasi-business transaction network utilizing data from 5224 Korean companies in the disaster safety industry. By aggregating firm-level data to the regional level, we can examine the patterns and roles of firm relationships within the disaster safety sector in Korea on a geographical scale. This study examines the spatial characteristics of the disaster safety industry and its association with regional industries. It begins by summarizing what the disaster safety industry is and its international implications. Next, the quasi-business transaction network of the industry is analyzed based on graph theory to identify its unique characteristics. To overcome the difficulty of detecting clusters of emerging industries, we examine the spatial characteristics of the transaction network through community detection algorithm. The study also identifies the relationship between the derived clusters and the location quotients of other existing industries within the clusters. Finally, the study highlights policy implications and future issues related to the disaster safety industry.

## 2 Literature review

### 2.1 Fostering the disaster safety industry in Korea

Korea's disaster safety industry was driven by demand from the government, resulting in an artificially established industry. Disaster and Safety Industry Promotion Act of Korea defines the disaster safety industry as an industry that develops, produces, distributes, or provides services related to technology, equipment, facilities, products, etc. to protect human life, body, and property from disasters or other accidents (article 2). The act (article 3) also mandates that both national and local governments must implement policies to support and promote the development of the disaster safety industry [16]. However, there is still a lack of agreement on identifying and classifying the disaster safety industry [6,17]. This is mainly due to the limitations of current industrial classification systems based on traditional activities- or supply-based systems, such as the North American Industry Classification System (NAICS) and the United Nations' International Standard Industrial Classification (ISIC) system [12]. In Korea, the Korea Standard Industrial Classification (KSIC) system was established in 1963 based on ISIC system. However, owing to advances in technology, this activity- or supplier- oriented industrial classification system hardly reflects the emerging industry and their hierarchical structure [18]. The demand-based industrial classification system has emerged as an alternative to this change [3]. Reflecting these alternatives, demand- or market-based classifications such as North American Product Classification Systems and Global Industry Classifications Standards have emerged. This system has developed a comprehensive and demand-based hierarchical aggregation system that can be linked to the existing supply-based classification systems. These industrial classifications provide a valuable framework for identifying and managing emerging industries.

In 2015, the Korean government developed a new demand-based industrial classification system for the disaster safety industry that can be linked to the existing KSIC [3]. However, there were a lot of criticism that the first Disaster and Safety Industry Classification (DSIC) system was ambiguous and did not reflect the characteristics of this industry [19]. In other words, the first DSIC system failed to establish a demand-based classification for the Korean disaster safety industry. To address this limitation, the Korean government revised the DSIC system by proposing a hazard and function-based approach to reflect the characteristics of the industry in 2018 [3]. According to the recent analysis, the revised classification system for the disaster safety industry was more successful than the first one in the viewpoint of the demand-based needs of the Korean government [3].

At the same time, the Korean government established the first Comprehensive Planning for Developing Disaster and Safety Management Technology under the Framework Act on the Management of Disasters and Safety in 2007. This served as an opportunity to emphasize the private sector's capabilities in Korean disaster safety industry. Finally, in 2022, the Disaster and Safety Industry Promotion Act was enacted, laying the legal foundation for fostering the disaster safety industry. These Acts and plans basically represent the Korean government's emphasis on science and technology in disaster safety industry. One of the primary strategies employed by governments to promote the growth of the disaster safety industry is through the establishment of regional clusters [15]. The objective is to develop specialized industry clusters in each region that closely aligns with the existing [20].

According to the 2021 Disaster Safety Industry Survey conducted by the Korean Ministry of the Interior and Safety, as of 2020, there are 64,141 businesses with 393,010 employees in this industry. Compared with the 2020 Korean Economic Survey [21], it accounted for 1.06% of the total number of businesses and 1.58% of the total number of employees in Korea. The total annual sales of the Korean disaster safety industry were $34 billion, with 92.7% of small and medium-sized enterprises (SMEs). In addition, when examining the transaction characteristics of disaster safety industry businesses, substantial number of sales destinations were found to be local governments and public institutions. Comprehensively, businesses in the disaster safety industry are mainly composed of SMEs and depend on demand in the public sector.

### 2.2 Emerging industry cluster

Discussions on industry clusters can be seen in Adam Smith's discussion of the division of labor and specialization [22], Marshall's theory of industrial districts [23], Scott and Storper's new industrial space [24], and Porter's diamond model. Marshall [23] developed an industrial district theory explaining the phenomenon in which companies accumulate and obtain an integrated economy at a spatial level, and Porter [8] systematized the cluster theory by modernizing and reinterpreting Marshall's theory. Potter [8,25] defines clusters as geographical aggregates of interrelated enterprises and institutions in a particular industry. Companies in clusters experience stronger growth and faster innovation than those outside clusters [26,27]. These characteristics make clusters a prerequisite for strengthening regional competitiveness [28,29].

Emerging industries are created by disruptive ideas that impact social acceptance and market demand [7] and generally refer to industries in the form of a combination of new and previous technologies [7,12]. Emerging industries tend to integrate innovation and creative opportunities spatially. The cluster life cycle theory explains that the development process of industrial clusters can show different development processes, even in homogeneous industrial clusters, because regional factors work in combination [13,26,30]. Porter [25] divided the dynamic flow of clusters into three stages: creation, evolution, and decline. During the creation period, clusters began to form, led by a small number of innovative companies based on the historical background of the region, local universities, demand reflecting regional characteristics, and the existence of related companies in the existing region. Self-reinforcing cycles occur in clusters during the evolutionary period, such as revitalizing the competition between local institutions and companies in the region, introducing high-quality human resources and knowledge, and activating related industries, resulting in growth and evolution. Menzel and Fornahl [13] divided the cluster lifecycle into four stages based on integration and employment growth rates. When clusters begin to form, the degree of integration within the range, businesses, and employment growth is not high, making it difficult to identify them as clusters. When the cluster company enters the growth stage, the degree of integration reaches the national level, shows a growth rate above the national level even though it is in the same industry, gradually increases to the network between companies and institutions, and is identified as a cluster.

Emerging industries, especially in the early stages of their life cycle, show a trend to collocate [10], and independent companies benefit from the presence of other companies [12,26]. Related industries integrate the technology and system of the existing industrial structure through interactions with emerging industries, and clusters develop [30]. Adopted technical specialization and business models can mimic local conditions, and related industries can provide a means for technological and industrial evolution [30]. Therefore, the region's existing industrial structure plays a decisive role in capturing and fostering emerging industries.

### 2.3 Business network analysis based on graph theory

Network analysis, underpinned by graph theory, forms the bedrock for investigating complex networks and their intrinsic properties. This interdisciplinary domain of study emerged from the rudimentary conception of graphs as a collection of vertices, interconnected by edges, representing nodal points and their pairings. Over time, mathematicians have elucidated a unique lattice structure inherent to these networks [31]. Consequently, graph theory has evolved into topological geometry, encompassing mathematical methodologies pertinent to network analysis. This advancement facilitates a coherent depiction of interconnections amongst social phenomena [32], while also enabling the quantitative assessment of their relationships [33,34]. Network analysis is a methodology for detecting, describing, and analyzing the relationships among organizations or firms [35]. It effectively represents structural relationships and quantifies them to explain their causes and consequences. Network analysis visually shows a network composed of nodes and edges, which allows for identification of important objects and clusters to enhance network efficiency or derive policy implications [36,37].

Recently, the importance of network analysis has increased as a means of understanding social phenomena [38], and many studies using network analysis have emerged, including business transaction network characteristics. Studies using business transaction analysis related with industry clusters can be categorized into two types: geographical and functional. Geographical cluster studies focus on regional industrial competitiveness. Mizuno et al. [39] analyzed the customer-supplier network structure of 500,000 Japanese companies and suggested that the correlation between the growth rate of companies and geographical characteristics was high. Jung [40] analyzed the network of business-to-business transactions in southeastern Korea. This study demonstrated the formation of industrial clusters centered on large corporations, closed transaction relations between industrial clusters, and the primary transaction phenomenon between distant regions. It can be inferred that these studies reflect the cluster characteristics presented by Porter [8] as geographical aggregation in regional transaction relationships. Choi [41] explored the digital content industry's institutional and geographical network structures in Korea. The results of the study confirmed that industries expanded outside metropolitan areas in the late 2000s. Marra et al. [42] investigated emerging green-tech companies in San Francisco, New York, and London to identify their specialization, emerging aggregates, and specific clusters. Based on metadata, they proposed a network analysis of technological innovations produced by green-tech firms.

Studies that derive functional clusters attempt to identify the potential of emerging industries demonstrating innovative functional synergy in and between the industries. Pekkarinen and Harmaakorpi [43] analyzed the network structure of a well-being industry innovation cluster in Finland. Giuliani [44] analyzed the knowledge network and business network structure of wine clusters in Italy. They explored the expansion and innovation processes of the cluster by focusing on the functions of emerging industry. In contrast, Cassetta et al. [6] and Kim and Kim [45] infer the process of creating new industries from existing industries. Cassetta et al. [6] employed a network analysis to detect emerging clusters of firms founded between 2001 and 2016. In the study, three clusters related to new mobility were identified with business transaction analysis. Kim and Kim [45] analyzed Korea's business transaction network to produce electric and fuel-cell vehicles. The study suggested that the business transaction network of eco-friendly vehicles is less central and weaker than that of internal combustion engine vehicles. This study indicates that market demand was reflected in the growth stage of the internal combustion engine vehicle industry cluster, and the eco-friendly vehicle industry was introduced into an emerging industry.

### 2.4 Location quotients (LQ)

The location quotients (LQ) are one of the most popular indicators for identifying industrial specialization in a given region [46]. LQs are sensitive to the level of industry aggregation and definition of regions and benchmarks [46]. The number of business establishments and employees was used to calculate the location quotients. In general, industry specialization shows that the industrial LQ is greater than 1.0 in a given region [47,48]. However, some studies have defined industry specialization more strictly [49,50]. They defined industry specialization as a LQ greater than 1.25 and employing 0.2% or more of the local labor force to identify a given region [49,50]. These characteristics make the location quotients useful for characterizing industrial specialization, such as clusters.

Some studies examined core competency of local industries by using LQ [48,51]. For example, Kim and Park [48] examined core competency of the cultural content industry suitable for the characteristics of local regions. Mo and Lee [51] revealed that Gwangju, one of the metropolitan cities in south Korea, and neighboring cities have complementary industrial structures because Gwangju's core competency of local industries coincides with the neighboring cities' non-core competency.

Industrial clustering positively affects regional economies by creating agglomeration economies, technological innovation, and diffusion. Therefore, many empirical studies have been conducted to analyze the synergistic effect of industrial agglomeration through industrial clustering identified by location quotients [46,47,[52], [53], [54]]. Carroll et al. [52] used location quotients to identify a potential cluster region of the transportation equipment industry in four states in the Midwestern United States. Some studies analyzed LQ longitudinally to identified that the effects of industrial clustering on local economic development [48,54]. Kim and Park [48] quantified trends of LQ (2007–2011) to examine the trends of location competitiveness of the character culture content industry by region, based on the number of business establishments, employees, and sales. Niyimbanira et al. [54] quantified LQ (2002–2017) using five-year interval employment data in the coastal metropolitan cities of South Africa. They identified that sub-industries in the manufacturing and service sectors were significant drivers of local economic development, while the creation of new technology and business did not guarantee economic development in some metropolitan cities of South Africa [54]. Pominova et al. [46] tested the stability of location quotients to identify industry specialization in small cities and found that location quotients are stable at population sizes of approximately 4100 or more. The studies focused on examining the location characteristics and competitiveness according to industrial agglomeration by region and examining the potential for regional economic growth and the feasibility of location selection based on the competitiveness of a specific industry.

## 3 Data collection

This study uses transaction network data of companies in the Korean disaster safety industry. The data are from the 2021 Disaster Safety Industry Statistics conducted by the Korean Ministry of Interior and Safety. A total of 5224 disaster safety companies were surveyed, and each company responded to the names and locations of the major suppliers and vendors. Of these 5224 companies, 2791 responded to the supplier's question (response rate: 53.4%), and 3905 responded to the vendor's question (response rate: 74.8%). Unclear answers were removed after the researchers examined the company name and regional location. The analysis proceeded through 2334 linked data points of the supplier and respondents and 2520 linked data points of the vendor and respondents. The administrative districts of location quotients were classified into 17 metropolitan regions and 228 districts according to the 2022 Korea governmental administrative classification. Among them, level of districts (*si*, *gun*, and *gu*) are administrative divisions used for local government. Based on the total employment numbers of the Korean administrative at the level of districts provided by the Korean government microdata, industrial location quotients for each district were established based on 19 industrial categories: A. Agriculture, forestry, and fishing; B. Mining; C. Manufacturing; D. Electricity, gas, steam, and air conditioning supply; E. Water, sewerage, waste management, materials recovery, and remediation activities; F. Construction; G. Wholesale and retail trade; H. Transportation and storage; I. Accommodation and food service activities; J. Information and communications; K. Financial and insurance activities; L. Real estate activities and renting and leasing; M. Professional, scientific and technical activities; N. Business facilities management and business support services; O. Public administration and defense and compulsory social security; P. Education; Q. Human health and social work activities; R. Arts, sports and recreation-related services; S. Membership organizations, repair, and other personal services.

## 4 Methodology

### 4.1 Business network analysis based on graph theory

Network analysis consists of data collection, analysis, and qualitative and quantitative interpretation [55]. Centrality analysis and community detection are typical methods used in network analysis, and network graphic representations and their interpretations are often performed using qualitative analysis methods [55]. This study utilized both quantitative and qualitative approaches to analyze the network structure of the disaster safety industry. The network was characterized using centrality analysis and uncertainty analysis based on graph theory. A community detection algorithm was also employed to detect subnetworks within the transaction network and identify potential clusters. A qualitative analysis was performed while visualizing a map using Geographic Information System (GIS) based on network centrality.

A network comprises nodes and edges. In this research, the nodes correspond to the regions embodying the companies incorporated in the analysis, while the edges illustrate the trade associations among these enterprises. During the data collection phase, every company within the disaster safety industry provided information concerning the names and regions of its suppliers and distributors. Nevertheless, it is noteworthy that this data set did not incorporate explicit details regarding transaction volumes. To expedite network analysis, each company was transposed to a specific administrative district within South Korea. The ensuing network was structured as a directed graph, in which an edge is assigned a weight of 1 if it denotes at least one trade relationship, and 0 if it does not.

Network centrality is a representative method widely used to identify influential nodes in a network [56]. The weighted degree centrality is the sum of all weights in which one node is connected to another node, and is proportional to the number of edges [57]. The high weighted degree of centrality can be seen as an area that deals with other districts frequently. In addition to centrality, the concept of a self-loop was used in this study. This is a value that connects one node to the same node, and in this analysis, it is interpreted as the frequency with which transactions occur within the district [56].

This analysis also assessed the robustness of the quasi-business transaction network under conditions of uncertainty. Due to the construction of networks in this analysis through geographic aggregation using firms' transaction relationships, there is a possibility of missing or hidden data. Therefore, it is crucial to consider the robustness of the network measurements used in the analysis to ensure the validity of the study [58,59]. Uncertainty in a network is typically evaluated by altering the values of nodes or edges [59]. As the nodes in this analysis represent uncontrollable administrative districts, edge weights (i.e., the number of transactions) were manipulated (from 10% to 30% of their original values) to examine the resulting changes in network structure and centrality. Network robustness can be evaluated by comparing the simulated values with the original values [59]. A robust network is characterized by its ability to maintain overall structure and functionality despite alterations, disruptions, or failures occurring at its edges [59]. The uncertainty analysis was conducted using R software.

Once the network is configured, it is also possible to find a subnetwork through community detection to identify the characteristics of the network [60]. The modular optimization community detection method is a representative agglomerative approach used to extract the community structure [55,60]. This method derives optimal modularity values, starting from a community composed of only one node and repeating the process of integration with other nodes [60]. The process was calculated using a fast unfolding algorithm based on the Laplacian method [60]. In this analysis, the process of deriving regional clusters of the business transaction network were derived through modularity analysis of Gephi software.^1^ The network characteristics, including reciprocity (Eq. (1)), global clustering coefficient (Eq. (2)), and network centrality indicators, were analyzed using Netminer 4.0.^2^ Global clustering coefficient ranges between 0 and 1, where a value of 1 indicates that all the nodes in the network are connected to each other, forming a complete graph. A value of 0 indicates that no triangles exist in the network. This analysis helped to recognize and understand the unique features of the network.

(1) $$Reciprocity=\frac{number\;of\;links\;pointing\;in\;both\;directions}{total\;number\;of\;links}=\frac{L↔}{L}$$

(2) $$Global\;Clustering\;Coefficient=\frac{3\times number\;of\;triangles}{number\;of\;trplets}=\frac{number\;of\;closed\;triplets}{number\;of\;trplets}$$

Network data are not random samples from a population, and each observation value is interdependent [61]. Therefore, the methods of general inference statistics cannot be directly applied to matrix-type data. To verify the statistical significance of the interconnected data, a separate test method, that is, a permutation test, is required [61,62]. Permutation refers to rearrangement, and the data rearranged in the network analysis is a matrix of network data [63]. The permutation test is a method of extracting countless samples through the bootstrap method under the assumption that the null hypothesis is correct, and performing a statistical significance test based on the sample distribution consisting of the statistics of these samples [62]. In addition to testing indicators within a network, statistical significance testing methods based on the concept of permutation can also be used to test the relationship between network indicators and node non-relational indicators such as population density. In this study, samples mimicking probability distributions were extracted, and significance was verified through Markov Chain Monte Carlo sampling methods.

### 4.2 Location quotients

The location quotients (LQ) are index that measures the relative specialization between regions of the industry, as shown in Eq. (3) by comparing the proportion of a specific industry to the total (national) proportion of the region. If the location quotients of a specific industry are greater than 1, it can be said that a specific industry in the region is specialized, and the higher the value, the greater the degree of specialization. However, in this study, an industry with location quotients of more than 1.25 was defined as a regionally specialized industry based on previous studies [49,50].

(3) $${LQ}_{ij}=\frac{\frac{Q_{ij}}{Q_{j}}}{\frac{Q_{i}}{Q}}=\frac{\frac{Regional\;Industry\;Employment}{Regional\;Total\;Employment}}{\frac{National\;Industry\;Employment}{National\;Total\;Employment}}$$

## 5 Results

### 5.1 Business network analysis

#### 5.1.1 Characteristics of quasi-business transaction network

Business transaction network is visualized based on the number of nodes and edges. The node size was expressed in proportion to the self-loop which means the volume of transactions within the district. Edge thickness was expressed in proportion to the weighted degree, including the direction of the two nodes. The network's reciprocity and global clustering coefficients were measured to determine the characteristics of the disaster safety industry network, and statistical significance was verified. As shown in Table 1, the reciprocity of the network was 0.396 (p < 0.001). The significance confirms that the pairwise transaction relationships in this network are greater than in the randomized networks (expected value: 0.243). In other words, the districts tended to trade closely with each other rather than unilaterally. The global clustering coefficient of the network was 0.458 (p < 0.001), which means that nodes in graph are more likely to tightly cluster together than in a randomized network (expected value: 0.435). The influence of pairwise transaction relationships on third-party nodes is also higher compared to randomized networks. Therefore, the transaction relationships between districts are relatively dense, forming tightly connected clusters in this network.

**Table 1:** Reciprocity and Clustering Coefficient of quasi-business transaction network.

Indicator	Observed	Expected	P > Obs	P=Obs	P < Obs	SD
Reciprocity***	0.396	0.243	0.000	0.001	0.999	0.027
Clustering Coefficient***	0.458	0.435	0.001	0.000	0.999	0.009

*p < 0.05, **p < 0.01, ***p < 0.001.

As a result of the analysis of the disaster safety industry network, weighted degree values, proportional to the transaction volume between districts, and self-loop, which represents the transaction volume within the district, were derived. A correlation analysis was conducted to determine the relationship between these indicators and the district's population density and gross regional domestic product (GRDP) (Table 2). As a result of the analysis, the correlation coefficient between weighted degree by district and GRDP in the district was 0.724 (p < 0.01), and the correlation coefficient with population density was 0.173 (p < 0.05). The correlation coefficient between self-loop, which represents the frequency of transactions in the district, and GRDP in the district was 0.551 (p < 0.01). The correlation coefficient with population density was 0.027, which was insignificant. The weighted degree and self-loop of the Korean disaster safety industry network were highly positively correlated with indicators representing regional demand.

**Table 2:** Correlation analysis results for Regional Indicators.

Regional Indicator	Weighted Degree	Self-Loop	Gross Regional Domestic Product	Population Density
Weighted Degree	1			
Self-Loop	0.893**	1		
Gross Regional Domestic Product	0.724**	0.551**	1	
Population Density	0.173*	0.027	0.218**	1

*p < 0.05, **p < 0.01, ***p < 0.001.

#### 5.1.2 Uncertainty analysis of network

**Fig. 1:**
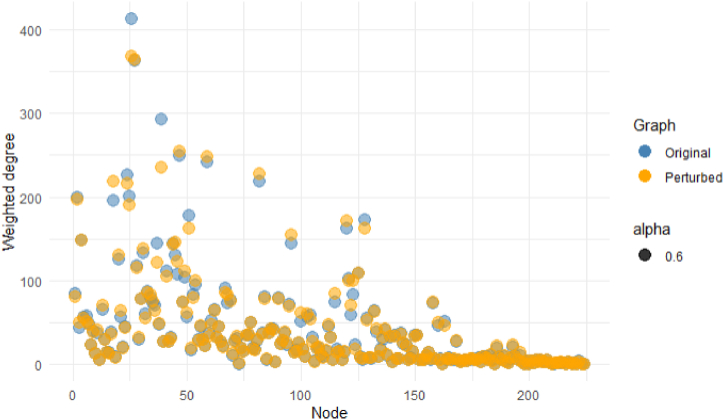
Difference in weighted degree between original and perturbed network (factor: 30%).

In this analysis, nodes (regions) are fixed because they are not affected by uncertainty. However, the number of transactions between regions (edges) may contain uncertainty due to measurement error, incomplete data, or other factors. Uncertainty is introduced into the network by randomizing edge weights and evaluating how the network structure and centrality respond to these changes. The robustness of the network is assessed based on centrality measures, specifically weighted degree, of the original and perturbed graphs. As shown in Table 3, a perturbation factor of 0.1, 0.2, and 0.3 is set, meaning edge weights are randomly perturbed by up to 30% of their original values. This simulates potential uncertainty in transaction data. By adjusting this factor, the robustness of the network under different levels of uncertainty is tested. When edge weights are perturbed by up to 30%, both the mean and median change by less than 10% (Fig. 1). This suggests that the business transaction network can withstand disruptions in uncertainty or transaction volume (edge weights) without significant changes in its structural characteristics.

**Table 3:** Summary statistics of weighted degree for each perturbation factor.

Perturbation Factor	Mean percentage change (%)	Median percentage change (%)	Standard deviation
10%	2.57	1.99	2.23
20%	4.98	3.75	4.38
30%	7.10	5.57	5.89

#### 5.1.3 Community detection of business transaction network

The community detection algorithm identified four clusters in the disaster safety industry network. In the community detection process, clusters are searched only through the weighted degree of each district. Fig. 1 shows the result of mapping the derived cluster to a GIS. Although it was not completely divided into administrative districts, clusters by region were classified to some extent through transaction relationships. Based on the name and map of the administrative district, communities including Seoul, Incheon, Gyeonggi-do, and Gangwon-do were named the Seoul Metropolitan Cluster (SMC), and communities including Busan, Gyeongsangnam-do, Gyeongsangbuk-do, and Jeju were named the Gyeongsang Cluster (GSC). Communities including Chungcheongnam-do, Chungcheongbuk-do, and Daejeon were named Chungcheong clusters (CCC), while Jeollanam-do, Jeollabuk-do, and Gwangju were named Jeolla clusters (JLC). Information on network indicators and population density for the major cities in each cluster is shown (Table 3).

**Fig. 2:**
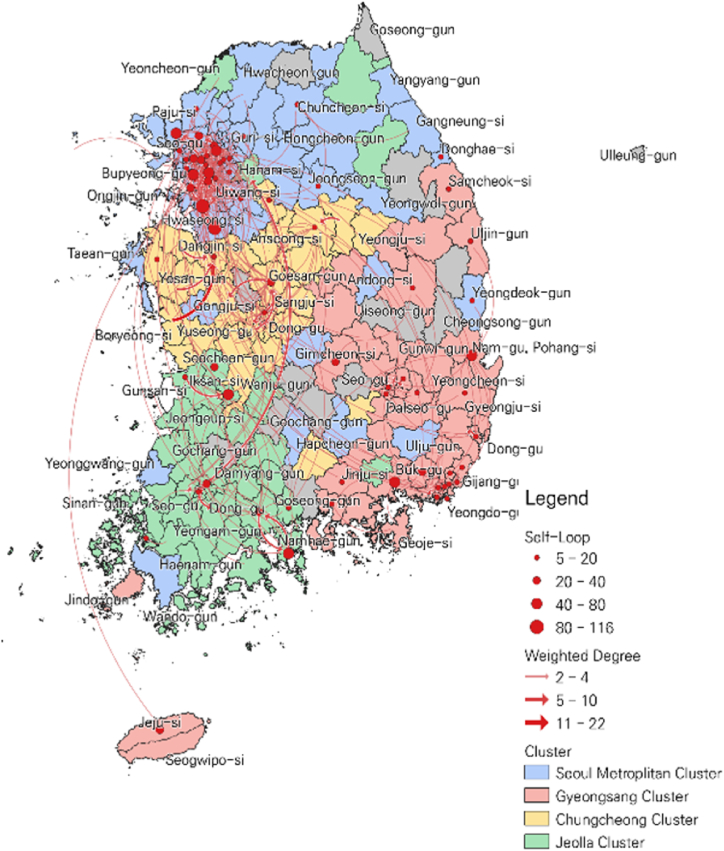
Cluster Mapping of Korean disaster safety industry transaction network.

As shown in Table 4, among the four clusters, the SMC cluster contained the most districts and exhibited the highest total weighted degree. This means that a large portion of disaster safety industry transactions occur mainly in the Seoul metropolitan area. In Fig. 2, it can be visually confirmed that many red points and red lines are concentrated in the metropolitan area. The GSC cluster, which includes the Gyeongsang region, has the second largest number of districts and shows relatively high average transactions frequency and average transaction within the district. The number of districts, including the CCC and JLC clusters, was similar. The JLC cluster showed relatively higher average transactions within the district and average total transaction frequency than the CCC cluster.

**Table 4:** Descriptive statistics on regional cluster of disaster safety industry network.

Cluster	Number of Node	Avg. Weighted Degree	Avg. Self-Loop	Avg. Total Weighted Degree
SMC	85	61.1	10.7	71.8
GSC	61	33.0	7.5	40.5
CCC	36	30.6	3.3	33.9
JLC	40	34.3	7.9	42.2

### 5.2 Location quotient (LQ)

#### 5.2.1 LQ of each cluster

For each cluster derived based on community detection analysis, location quotients for each cluster according to the KSIC were derived from examining which industries specialize in the existing region (Table 5). Location quotients were derived from data on the number of workers in 222 of the 228 districts where the business's transactions occurred. According to previous studies, when the location quotients exceeded 1.25, it was interpreted that the industry in the region was specialized [49,50]. First, it was found that the information and communication and professional, scientific, and technical activities industries specialized in the SMC cluster. In the CCC cluster, the manufacturing industry was found to be specialized. In the JLC cluster, it was found that electricity, gas, steam, and air conditioning supply; water supply; waste management, materials recovery; construction; and public administration and defense industries were specialized. It can be seen that different industries specialize in each cluster.

**Table 5:** LQ of regional clusters by Korea Standard Industrial Classification (KSIC).

Location Quotients	SSC	GSC	CCC	JLC
C. Manufacturing	0.833	1.224	1.416	0.907
D. Electricity, gas, steam and air conditioning supply	0.703	1.207	1.112	1.524
E. Water supply; sewage, waste management, materials recovery	0.798	1.107	1.324	1.363
F. Construction	0.95	0.994	0.963	1.311
H. Transportation and storage	1.008	1.052	0.895	0.995
J. Information and communication	1.538	0.375	0.426	0.404
K. Financial and insurance activities	1.098	0.93	0.742	0.991
M. Professional, scientific and technical activities	1.337	0.582	0.79	0.523
N. Business facilities management and business support services	1.195	0.793	0.853	0.69
O. Public administration and defence; compulsory social safety	0.839	1.009	1.135	1.294
P. Education	0.976	1.002	1.027	1.069
Q. Human health and social work activities	0.976	1.047	0.988	1.238

#### 5.2.2 Correlation analysis between weighted degree and location quotients

A correlation analysis was conducted to examine the relationship between the specialized industry of each cluster and the weighted degree of the disaster safety industry (Table 6). The analysis showed that the weighted degree of each district in the SMC cluster was correlated with industries with manufacturing (p < 0.01), professional, scientific, and technical activities (p < 0.05), and business facilities management and business support services (p < 0.05). The weighted degree of each district in the GSC cluster was highly correlated with professional, scientific, and technical activities (p < 0.05). The weighted degree of each district in the CCC cluster was highly correlated with the business facilities management and business support services (rental and relaxing activities) industries (p < 0.05). The weighted degree of each district in the JLC cluster was highly correlated with Transporting and storage (p < 0.05); financial and insurance activities (p < 0.05); professional, scientific, and technical activities (p < 0.05); and business facilities management activities (p < 0.05). In the remaining clusters, except for the CCC cluster, there was a positive correlation between professional, scientific, and technical activities and the weighted degree of the disaster safety industry network, indicating that there is a strong association between professional, scientific and technical activities, and disaster safety industry industries. In the remaining clusters, except the GSC cluster, there was a positive correlation between business facilities management and business support services and the weighted degree of the disaster safety industry network, indicating that there is a significant association between business facilities management and business support services and disaster safety industry industries. Table 7 shows the descriptive statistics on district's information by weighted degree.

**Table 6:** Correlation analysis results between weighted degree and LQs by regional cluster.

Correlation Analysis	SSC Weighted Degree	GSC Weighted Degree	CCC Weighted Degree	JLC Weighted Degree
C. Manufacturing	.353**	.142	−.021	−.122
D. Electricity, gas, steam and air conditioning supply	−.149	−.067	.072	−.087
E. Water supply; sewage, waste management, materials recovery	−.257*	−.090	−.106	−.263
F. Construction	−.205	−.088	.027	.273
H. Transportation and storage	−.053	.055	.065	.431*
J. Information and communication	.168	.143	.025	.037
K. Financial and insurance activities	.155	−.039	.167	.322*
M. Professional, scientific and technical activities	.243*	.265*	.096	.294*
N. Business facilities management and business support services	.215*	.174	.403*	.554**
O. Public administration and defence; compulsory social safety	−.420**	−.226	−.370*	−.553**
P. Education	−.268*	−.041	.043	.132
Q. Human health and social work activities	−.375**	−.208	−.142	−.038

*p < 0.05, **p < 0.01, ***p < 0.001.

**Table 7:** Descriptive statistics on district's information by weighted degree.

Seoul Metropolitan cluster (SMC)			Gyeonsang Cluster (GSC)			Chungcheong Cluster (CCC)			Jeolla cluster (JLC)		
District	Weighted Degree	Population Density $\left({km}^{2}\right)$	District	Weighted Degree	Population Density $\left({km}^{2}\right)$	District	Weighted Degree	Population Density $\left({km}^{2}\right)$	District	Weighted Degree	Population Density $\left({km}^{2}\right)$
Anyang	484	9533.5	Changwon	296	1389.9	Cheongju	194	905.6	Jeonju	262	3225.2
Hwaseong	448	1217.1	Pohang	200	444.5	Dadeok-gu (Daejeon)	111	2593.9	Buk-gu (Gwang ju)	200	4419.2
Pyeongtaek	409	1088.7	Gimcheon	103	139.3	Asan	105	639.6	Yeosu	186	3743.3
Siheung	305	3787.2	Jeju	97	499.2	Chungju	100	220.7	Suncheon	129	3604.8
Jung-gu (Seoul)	290	13144.6	Gimhae	96	1189.9	Seo-gu (Daejeon)	77	5049.4	Seo-gu (Gwang ju)	121	2128.9
Gimpo	259	1595.4	Gangseo-gu (Busan)	85	709.5	Boryeong	58	168.6	Iksan	114	1907.5
Jongno-gu	258	6464.7	Sasang-gu (Busan)	85	6150.3	Dangjin	53	241.1	Gunsan	99	1661.7
Ansan	238	4784.2	Nam-gu (Ulsan)	84	4511.9	Cheonan	52	1068.4	Gwangyang	79	873.4
Guro-gu (Seoul)	209	21696.3	Dalseo-gu (Daegu)	78	9142.4	Icheon	50	483.2	Mokpo	79	745.5
Gunpo	171	7554.3	Yeonje-gu (Busan)	74	16816.3	Yuseong-gu (Daejeon)	41	2083.2	Gwangju	71	684.9

## 6 Discussion

In this investigation, the disaster safety sector was structured as a district-level business transaction network. To discern the salient features of this network, metrics such as reciprocity and global clustering coefficients were employed, alongside the execution of uncertainty analysis grounded in graph theory. The subsequent business network analysis revealed that firms within the disaster safety domain exhibit greater interconnectivity and have a propensity to form compact clusters, in contrast to randomly generated networks. These observations corroborate existing research, which posits that nodes in real-world networks demonstrate heightened interconnectivity [56].

Furthermore, an uncertainty analysis was undertaken to ascertain the robustness of the network in question. Given that the nodes represent districts, edge perturbations (i.e., transaction volumes) were introduced in three distinct scenarios. Following a perturbation of up to 30%, the average mean and median percentage alterations persisted within a 10% range, suggesting that the network is capable of preserving its overarching structure and functionality in spite of alterations, disruptions, or edge failures [59]. These results can also provide reliability, especially when identifying sub-networks and interpreting the findings.

One characteristic of emerging industries is their drive by social demand [64]. In the context of the Korean disaster safety industry network, the weighted centrality index exhibits a positive correlation with both population density and Gross Regional Domestic Product (GRDP), signifying social demand. Consequently, regions with elevated population and economic standing experience heightened activity within disaster and safety-related sectors. This observation underscores the necessity of devising policies that account for the distinct characteristics inherent to these industries.

The pre-existing industrial structure of a region plays a pivotal role in fostering and nurturing emerging industries [30]. As such, it is imperative to investigate the interplay between these nascent industries and those already specialized within a given region. The results shows that it is evident that distinct industries specialize in each regional cluster. For instance, professional, scientific, and technical activities demonstrate specialization exclusively in metropolitan areas. In contrast, the Gyeongsang and Chungcheong regions exhibit a relative specialization in the manufacturing industry. Nonetheless, upon analyzing the correlation between the disaster safety industry network and extant regional location quotients, a positive association with professional, scientific, and technical activities emerged as a commonality in the SSC, CCC, and JLC clusters. This finding indicates that, contrary to existing literature on demand-based industries [4], innovation driven by science-based industries is already underway within the Korean disaster safety sector.

## 7 Conclusion

Cultivating the disaster safety industry is crucial for enhancing disaster resilience and competitiveness. However, the private sector faces challenges in developing this industry without government intervention, as transaction characteristics of the disaster safety industry predominantly stem from public demands. Consequently, policies are required to actively promote the disaster safety industry under governmental leadership. One strategy involves accurately identifying regional bases and actively fostering regional clusters. By implementing differentiated policies tailored to specific regional clusters, more efficacious outcomes can be achieved.

However, many emerging knowledge-intensive industries are difficult to capture using traditional industry classifications [12]. Standard classification codes based on supply, including SIC, NAICS, and KSIC, delineate industry clusters that have attained maturity within their respective life cycles. The spatial distribution of employment in these sectors pertains to regional hubs of production and employment yet does not align with the concentration and growth patterns observed in these nascent industries. Alternative approaches are requisite for the identification and comprehension of such emerging sectors. Cluster identification via community detection methods is determined solely through transaction relationships and does not require any other prior information or assumptions [65,66]. Upon identifying clusters in the disaster safety industry network using cluster identification method, a geographic concentration was observed surrounding major cities. The agglomeration of clusters discerned within regional units was deemed statistically significant through the employment of the global clustering coefficient. Taking into account the geographical proximity and spatial distribution among industrial clusters, numerous instances can be regarded as a single cluster when viewed from a broader regional perspective.

Innovation, which generates added value through market shifts as a consequence of technological development, can be achieved by two strategies: technology push and market pull. From the technology push standpoint, emerging industries focus on enhancing productivity through novel products and services derived from extant industries. In contrast, the market pull approach posits that growth arises from the demands of the private sector or government. From its inception, Korea's disaster safety industry has closely aligned with the market pull approach, driven by government needs—a finding consistent with the correlation analysis between network indicators and regional demand indicators. However, upon examining the relationship between the existing industry and the disaster safety industry, a strong correlation with science and technology-related industries emerged.

This observation suggests that policies enacted by the Korean government to stimulate science and technology innovation, spanning from the first to third Comprehensive Planning for Developing Disaster and Safety Management Technology, have exerted a tangible impact on the market. In essence, as an emerging industry, Korea's disaster safety sector exhibits unique characteristics, displaying both technology push and market pull attributes, which ought to be taken into account when devising future nurturing policies.

This study acknowledges several limitations. Firstly, the metrics utilized in this study are approximated at a regional level, thereby potentially confining their interpretation for the identification of comprehensive industrial trends. Secondly, in constructing a business transaction network derived from transaction relationships, it was not possible to weight the transferred quantity, irrespective of whether it was measured in terms of products, services, or monetary units. While the Korean government has been proactive in nurturing and managing the disaster safety industry, there remains an absence of precise quantitative information pertaining to transaction volumes segmented by region. Consequently, there is a need for a more detailed analysis of the network at a micro-level in future research. Finally, it is important to note that the network examined in this study represents only the transaction relationships and may not capture the complete characteristics of the entire disaster safety industry. Real-world networks frequently incorporate intangible features, including shared research initiatives, strategic alliances, and joint applications of technological patents, aspects that have been comprehensively addressed in preceding studies. Thus, future research endeavors should concentrate on fabricating a network that encapsulates these crucial intangible characteristics pertinent to Korean disaster safety industry enterprises to attain a superior understanding of their unique traits.

## Author contribution statement

1) conceived and designed the experiments: Ji-Bum Chung
2) performed the experiments: Min-Kyu Kim
3) analyzed and interpreted the data; Min-Kyu Kim, Jungseok Seo
4) contributed reagents, materials, analysis tools or data; Min-Kyu Kim
5) wrote the paper: Min-Kyu Kim, Jungseok Seo, NaYoon Kim, Ji-Bum Chung

## Data availability statement

Data will be made available on request.

## Additional information

No additional information is available for this paper.

## Declaration of competing interest

The authors declare that they have no known competing financial interests or personal relationships that could have appeared to influence the work reported in this paper.

## Footnotes

1 Gephi is an open-source network exploration and manipulation software. In this study, the Gephi software was used for detecting communities in the networks by conducting modularity analysis.

2 Netminer 4.0 is a commercial network analysis software that provides statistics and machine learning algorithm along with network analysis. In this study Netminer 4.0 was used for data preprocessing, network analysis and correlation analysis with permutation test.
